# Tissue-Specific Expression of DNA Methyltransferases Involved in Early-Life Nutritional Stress of Chicken, *Gallus gallus*

**DOI:** 10.3389/fgene.2017.00204

**Published:** 2017-12-06

**Authors:** Seong W. Kang, Mahmoud Madkour, Wayne J. Kuenzel

**Affiliations:** ^1^Department of Poultry Science, Center of Excellence for Poultry Science, University of Arkansas, Fayetteville AR, United States; ^2^Department of Animal Production, National Research Center Giza, Egypt

**Keywords:** nutritional stress, corticosterone, liver, anterior pituitary, DNA methyltransferase, passive demethylation

## Abstract

DNA methylation was reported as a possible stress-adaptation mechanism involved in the transcriptional regulation of stress responsive genes. Limited data are available on effects of psychological stress and early-life nutritional stress on DNA methylation regulators [DNMTs: DNA (cytosine-5)-methyltransferase 1 (DNMT1), DNMT1 associated protein (DMAP1), DNMT 3 alpha (DNMT3A) and beta (DNMT3B)] in avian species. The objectives of this study were to: (1) investigate changes in expression of DNMT1, DMAP1, DNMT3A, and DNMT3B following acute (AS) or chronic immobilization stress (CS); (2) test immediate effect of early-life nutritional stress [food deprivation (FD) for 12 h (12hFD) or 36 h (36hFD) at the post-hatching period] on expression of DNA methylation regulators and glucocorticoid receptor (GR), and the long-term effect of early-life nutritional stress at 6 weeks of age. Expression of DNMTs and plasma corticosterone (CORT) concentration decreased by CS compared to AS (*p* < 0.05), indicating differential roles of DNA methylation regulators in the stress response. Plasma CORT at 12hFD and 36hFD birds increased compared to control birds (12hF and 36hF), but there were no significant differences in plasma CORT of 12hFD and 36hFD birds at 6 weeks of age compared to 6 week controls. DNMT1, DMAP1, and DNMT3B expression in the anterior pituitary increased by 12hFD, but decreased at 36hFD compared to their controls (*P* < 0.05). In liver, DNMT1, DNMT3A, and DNMT3B expression decreased by 12hFD, however, no significant changes occurred at 36hFD. Expression of DMAP1, DNMT3A, and DNMT3B in anterior pituitary and DMAP1 and DNMT3A expression in liver at 6 weeks of age were higher in 36hFD stressed birds compared to controls as well as 12hFD stressed birds. Hepatic GR expression decreased by 12hFD and increased by 36hFD (*p* < 0.05). Expression patterns of GR in the liver of FD stress-induced birds persisted until 6 weeks of age, suggesting the possible lifelong involvement of liver GR in early-life nutritional stress response of birds. Taken together, results suggest that DNA methylation regulator genes are tissue-specifically responsive to acute and chronic stress, and hepatic GR may play a critical role in regulating the early-life nutritional stress response of birds. In addition, the downregulation of DNMT1 and DMAP1 may be one of the adaptive mechanisms to chronic early-life nutritional stress via passive demethylation.

## Introduction

Early-life stress can impact later health and growth performance by maladaptation of the stress-response system (Dallman, 1993; Maniam et al., 2014; Dixon et al., 2016). Nutritional stress in food animals is a critical issue especially in poultry species during specific periods of their life cycle, and was implicated by data suggesting epigenetic–genomic interactions (Gonzalez-Recio et al., 2015; Murdoch et al., 2016). A current issue in food animal production is the need for an objective measure to determine when an acute stress (AS) becomes chronic. Chronic nutritional stress appears to have a more detrimental effect on growth performance in food animals (Juul-Madsen et al., 2004). It is well recognized that DNA methylation is a major epigenetic factor influencing gene activities (Moore et al., 2013; Jeltsch and Jurkowska, 2014). Epigenetics can be defined as inheritable and reversible phenomena that affect gene expression without altering the underlying base pair sequence (Choi et al., 2013). One example is the influence of nutrients on epigenetic phenomena such as DNA methylation that have been extensively investigated (Choi et al., 2013).

DNA methylation was reported as a possible stress-adaptation mechanism involved in the transcriptional down-regulation of specific genes activated by stress (Murgatroyd et al., 2009; Mifsud et al., 2011; Li and Zhang, 2014; Schubeler, 2015; Ludwig et al., 2016; Kim and Costello, 2017). One of the best-characterized epigenetic changes is DNA methylation by the addition of a methyl group to DNA, thereby often modifying the function of the genes (Li and Zhang, 2014). CpG islands were focused on early studies on DNA methylation study (Wan et al., 2015). The DNA methylation process in the CpG islands is the addition of the methyl group at the 5-carbon of the cytosine ring resulting in 5-methylcytosine (5mC), and this methyl groups project into the major groove of DNA and inhibit transcription (Moore et al., 2013). CpG islands have a propensity to co-localize with the transcription start sites (TSS) of genes, and importantly the promoter CpG islands of genes are usually unmethylated (Bird, 2002; Ginno et al., 2012). The DNA methylation in CpG islands is primarily associated with decreased gene expression and is important for tissue-specific gene regulation (Wan et al., 2015). Recent studies provide a basic picture of the avian methylome (Head, 2014). A genome-wide mapping of DNA methylation patterns from liver and muscle from week-old chicks showed characteristics of the classic vertebrate patterns of DNA methylation, comprising unmethylation of CpG islands in gene promoters and enrichment and repetition in gene bodies, showing that DNA methylation is associated with decreases in transcription in birds (Head, 2014). DNA methylation is catalyzed by a family of DNA methyltransferases (DNMTs) that transfer a methyl group from *S*-adenyl methionine (SAM), the major methyl donor for most methyltransferase reaction in cells, to the fifth carbon of a cytosine residue to form 5mC. DNMT 3 alpha (DNMT3A) and 3 beta (DNMT3B) can establish a new methylation pattern to unmodified DNA and are thus known as *de novo* DNMT (Chen and Riggs, 2011; Baubec et al., 2015). On the other hand, DNMT1 functions during DNA replication to copy the DNA methylation pattern from the parental DNA strand onto the newly synthesized daughter strand (Wu and Zhang, 2010; Moore et al., 2013). DNA methylation can contribute to transcriptional silencing through several transcriptionally repressive complexes, which include methyl-CpG binding domain proteins and histone deacetylase (HDAC) (Rountree et al., 2000). DNMT1 protein can form a repressive transcription complex and the non-catalytic terminus of DNMT1 interacts to HDAC2 and DNMT1 associated protein (DMAP1), which has intrinsic transcriptional repressive activity (Rountree et al., 2000; Muromoto et al., 2004).

Glucocorticoids (GCs) are well known to regulate hepatic gene expression for control of energy metabolism in response to stress. Hepatic gene expression including glucocorticoid receptor (GR) and insulin-like growth factor 1 (IGF-1) is negatively correlated with serum corticosterone (Cheng et al., 2004). Growth hormone appears to modulate gene expressions of DNMT1 and DNMT3A in the Ames dwarf mouse liver tissue and primary hepatocytes, suggesting that GH deficiency may contribute to epigenetic stability by decreasing DNMT1 (Armstrong et al., 2014). However, in avian species, limited data are available on the effects of chronic immobilization stress (CS) and early-life nutritional stress on DNA methylation regulators (DNA methyltransferases and associated protein) related to impairment of the hypothalamic-pituitary-adrenal (HPA) axis and maladaptation of the stress-response. The current study was designed to test the hypothesis that changes in DNA methylation regulators and GR gene expression are dependent on the stress status (acute vs. chronic). We investigated changes in DNMTs and DMAP1 expression following acute or CS, and tested the immediate effect of early-life nutritional stress (food deprivation (FD) for 12 h or 36 h post-hatching) on DNMTs, DMAP1, and GR expression after treatment. Additionally, it was determined whether or not a long-term effect of early-life nutritional stress occurred at 6 weeks of age.

## Materials and Methods

### Ethics Statement

The care and experimental use of animals were approved by the University of Arkansas Institutional Animal Care and Use Committee (Protocol # 16043). Animals were maintained according to a standard management program at the Poultry Farm, University of Arkansas.

### Animals and Sample Preparation

Experiment 1: One-day old male chicks (Cobb 500) were obtained from a commercial hatchery, transported to laboratory facility, and raised in an environmentally controlled room. A standard commercial starter diet was fed *ad libitum*. Chicks were maintained on continuous light (L) with no dark (D) periods (LD 24:0) for 3 days in order for the birds to find and recognize food and water. Birds were then transferred to a long-day photoperiod of LD 16:8 for the remainder of the study. At 2-weeks of age, birds were randomly assigned to four treatment groups (*n* = 8): acute control (AC), AS, chronic control (CC) and chronic stress (CS). All birds were kept individually in cages under long-day photoperiod (lights on 07:00 h) until 6 weeks of age. Immobilization (restraint) stress was applied to birds as a psychological stressor to study neuroendocrine regulation of avian HPA axis in our previous studies (Kang and Kuenzel, 2014; Nagarajan et al., 2014: Aman et al., 2016). AS was induced for 1 h of immobilization by wrapping each bird in a harness that prevented birds from standing or moving their upper bodies including their wings. Each bird had access to water as previous described (Selvam et al., 2013; Jayanthi et al., 2014; Kang and Kuenzel, 2014). To minimize human presence and handling stress, sampling of blood from each bird was consistently and gently performed by the same person who cared for and managed the birds throughout the study. Blood was sampled from the wing vein (brachial vein) before and after 1 h acute immobilization stress. AC birds were not exposed to immobilization stress and blood was sampled after 1 h without immobilization stress. Birds chronically stressed were exposed to 1 h immobilization for 10 consecutive days when 6 weeks of age. Blood was sampled on day 10 after 1 h stress. For the CC treatment group, blood was sampled on day 10 without any previous stress treatment. At the end of stress treatments, in addition to blood sampling, anterior pituitary glands were collected and stored at 4 and -80°C, respectively.

Experiment 2: One-day old male chicks from a commercial hatchery were transported to our laboratory facility. Chicks were randomly divided into five treatment groups (*n* = 18/treatment): The first group was designated zero time point controls, which was sampled immediately at 0 h (Con). A second group had free access to food and water for 12 h and sampled as 12 h controls (12hF). A third group was sampled at 36 h after continual access to food and water and designated 36 h controls (36hF). A fourth and fifth group were kept for 12 and 36 h without food and water, respectively, and sampled as the 12 h FD group (12hFD), and 36 h FD group (36hFD). For sampling of the five treatment groups, chicks were rapidly decapitated and 1 ml blood was collected in heparinized tubes and stored at 4°C. The anterior pituitary and liver were dissected and snap frozen in dry ice, and stored at -80°C. Blood samples were centrifuged and plasma was stored at -20°C until quantified for corticosterone (CORT) by radioimmunoassay (RIA).

Experiment 3: Three treatment groups were used for experiment 3 (*n* = 18/treatment). The first group had free access to food and water for 6 weeks and designated as controls (Con2). The second and third groups were kept for 12 h or 36 h without food and water, and thereafter birds had access food and water until 6 weeks of age and designated as the 12hFD2 and 36hFD2 group. At the end of the experiment, blood, anterior pituitary, and liver were sampled from each bird. Anterior pituitary and liver were dissected and snap frozen in dry ice, and stored at -80°C. Blood samples were centrifuged and plasma was stored at -20°C until assayed for CORT by RIA.

### RNA Isolation and Real-Time Quantitative RT-PCR

Total RNA was extracted from quick-frozen anterior pituitary or liver tissues using TRIzol^®^ reagent (Invitrogen Life Technologies, Palo Alto, CA, United States) followed by DNase I treatment and purification of total RNA by the RNeasy mini kit (Qiagen, Valencia, CA, United States). The RNA quality and quantity were determined using agarose gel electrophoresis and NanoDrop 1000 (Thermo Scientific, Wilmington, DE, United States). Two μg of total RNA from anterior pituitary glands or liver were converted into cDNA with oligo (dT)_16_ primer and SuperScript III reverse transcriptase (Invitrogen, Grand Island, NY, United States), as previously described (Jayanthi et al., 2014; Kang and Kuenzel, 2014; Nagarajan et al., 2017). The specific oligonucleotide primers were designed using the PRIMERS3 program^1^. Four primer sets for each gene were designed and performed conventional RT-PCR for optimizing annealing temperature for each primer set (**Table 1**). The PCR products were analyzed by using agarose gel electrophoresis (3%). Melting curve analysis and PCR efficiency for each selected primer set were validated with the default settings on the ABI 7500 system (Applied Biosystems LLC, Foster City, CA, United States). The efficiency of PCR was evaluated by performing a dilution series experiment and the slope of standard curve was translated into an efficiency value. Efficiency of the PCR within 96–100% was accepted for this study. A portion (1 μl) of the cDNA was subjected to quantitative real-time PCR (qRT-PCR) using an ABI 7500 system with Power SYBR Green PCR Master Mix (Invitrogen, Grand Island, NY, United States).

**Table 1:** Primers used for RT-qPCR.

Gene	GenBank#	Primer sequences (5′–3′)	Size (bp)	Annealing temperature (°C)
DNMT1	NM_206952	F:CATCCTCAGGGACCACATCT	169	58
		R: CTTCTTCTCGTGGTGGGTGT		
DMAP1	XM_004936716	F: CTTTGTGCTGCCTACTGCTG	151	58
		R: AGTTCTGCTCCTTGCTTTGC		
DNMT3A	NM_00102483223	F: CACCACTCGCTCCAACTCCA	141	60
		R: ATGTTGGACACGTCCGTGT		
DNMT3B	NM_00102482833	F: CTGCACACAGAGCTCGCTAC	167	60
		R:GAGAGGGGAGAAGAGGTGCT		
GR	NM_001037826	F: GCCATCGTGAAAAGAGAAGG	95	54
		R: TTTCAACCACATCGTGCAT		
GAPDH	NM_204305	F: CTTTGGCATTGTGGAGGGTC	128	58–60
		R: ACGCTGGGATGATGTTCTGG		
β-actin	L08165	F: CACAATGTACCCTGGCATTG	158	54–56
		R: ACATCTGCTGGAAGGTGGAC		


The conditions of real-time qRT-PCR were 1 cycle at 95°C for 5 min, 40 cycles at 95°C for 30 s, 56–60°C for 1 min. Chicken glyceraldehyde 3-phosphate dehydrogenase (GAPDH) and β-actin were used as internal controls. Dissociation curves were constructed at the end of amplification for validating the quality of the data. All qRT-PCR experiments were performed in triplicate and the values of the average cycle threshold (*C*t) were determined and Delta-*C*t scores for gene transcripts in each sample were normalized using Delta-*C*t scores for GAPDH/β-actin and expressed as the fold change in gene expression using the equation, 2^-ΔΔ*C*_T_^. The Gene name, NCBI accession numbers, primer sequences, PCR product size, and annealing temperatures used in the present study are shown in **Table 1**.

### Radioimmunoassay (RIA) of Plasma CORT

Plasma CORT levels were determined by RIA (Kuenzel et al., 2013; Kang and Kuenzel, 2014; Aman et al., 2016). The primary antibody against CORT was purchased from Fitzgerald Inc. (Concord, MA, United States) while the secondary antibody and ^125^I CORT tracer were purchased from MP Biomedicals Inc. (Orangeburg, NY, United States). The intra- and inter-assay coefficients of variation were 9 and 14%, respectively.

### Statistical Analyses

Statistical analyses were performed using JMP^®^ 11.0 (SAS Institute Inc., NC). A normal distribution was first tested and subsequently differences among the groups were analyzed using one-way analysis of variance (ANOVA) followed by mean comparison using the Tukey’s HSD test at a significance level of *p* < 0.05. Multiple comparisons of group means by Tukey’s HSD test were used to evaluate relative changes of gene expression among treatment groups for each gene. Data are presented as the mean ± SEM. A probability level of *p* < 0.05 was considered as statistically significant.

## Results

### Effects of Immobilization or Nutritional Stress on Plasma Corticosterone

**FIGURE 1:**
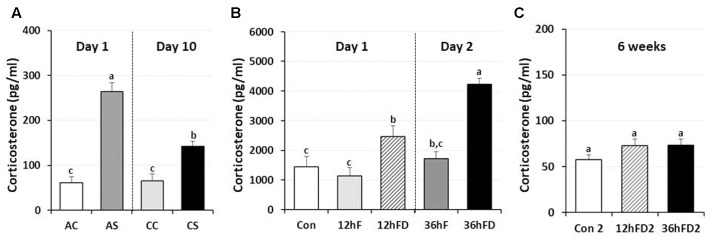
Differential response of plasma corticosterone (CORT) following acute, chronic immobilization stress (CS) or early-life nutritional stress in birds. **(A)** Attenuation of CORT response in chronic stressed birds compared to acute stressed birds by immobilization. Plasma CORT concentrations (pg/ml) were measured by radioimmunoassay (RIA) after acute or CS (*n* = 7–8 birds/group). **(B)** Plasma CORT concentrations were measured by RIA (*n* = 18 birds/group, duplicate). Treatments of 12 h (Day 1) or 36 h (Day 2) FD after post-hatching, blood was sampled from 0 h controls (Con), 12 h fed controls (12hF), 12 h FD (12hFD), 36 h fed controls (36hFD), and 36 h FD birds (*n* = 14birds/group). **(C)** At 6 weeks of age, blood was sampled from birds which were treated by 12hFD and 36hFD following post-hatching (Days 1 and 2). Plasma CORT concentrations were measured by RIA. Data (mean ± SEM) are presented as the fold changes of relative expression levels compared to the unstressed control group (Con) set at a value of 1.0. Different lower-case letters above the bars denote significant differences (*P* < 0.05) among groups, where a > b > c and b,c is not different from b or c.

In male chickens, 6–7 weeks of age, after 1 h acute immobilization stress (AS), plasma corticosterone (CORT) levels increased three–fourfold compared to unstressed birds (AC) as previously reported (Kuenzel et al., 2013; Selvam et al., 2013). Chronic immobilization stressed birds (CS) following 10 consecutive days of 1 h stress showed a 46% reduction in stress-induced CORT compared to CORT levels of acute stressed birds (AS) (*p* < 0.05, **Figure 1A**).

Nutritional stress for 12 h or 36 h FD (12hFD and 36hFD) increased plasma CORT by 110 and 140% compared to their controls (12hF and 36hF), respectively (**Figure 1B**). Nutritional stress during the neonatal period (Days 1 and 2 of age) for 12 h or 36 h did not significantly affect subsequent CORT levels when measured at 6 weeks of age (**Figure 1C**). However, there were slight increases of CORT by 26 and 27% in the 12 h FD and 36 h FD groups, respectively, compared to controls (Con2).

### Effects of Acute or Chronic Immobilization Stress on the Expression of DNA Methylation Regulators in the Anterior Pituitary

**FIGURE 2:**
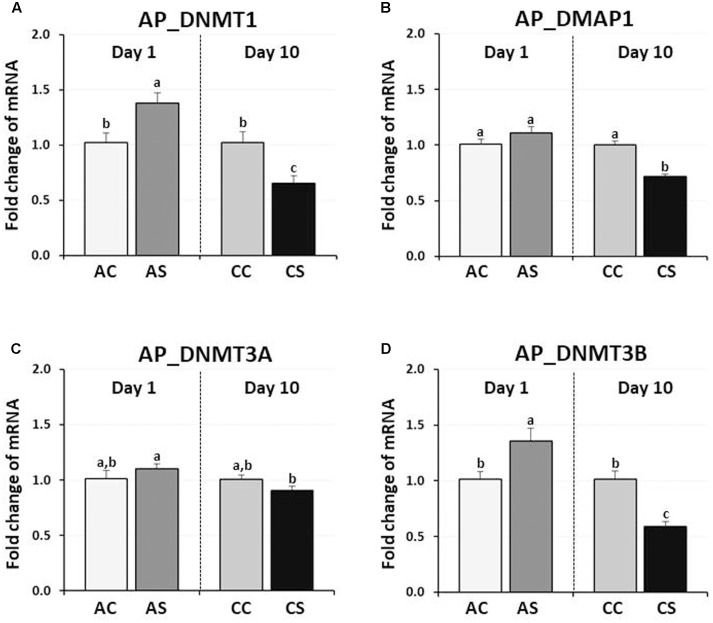
Differential changes in expression of DNA methyltransferases (DNMT1, DNMT3A, and DNMT3B) and DNMP1 in the anterior pituitary gland by acute or CS. Total RNA from the anterior pituitary was extracted after 1 h acute and after 10 consecutive days of 1 h CS. Relative mRNA levels of DNA methyltransferases were quantified and data were set as fold changes of relative expression levels using the ΔΔ*C*t method with GAPDH and β-actin as internal controls. Data (mean ± SEM) were expressed from a value set for 1.0 for the (AC) for each gene. Different lower-case letters above the bars denote significant differences (*P* < 0.05) among groups, where a > b > c and a,b is not different from a or b. DNMT1, DNA methyltransferase 1; DMAP1, DNA methyltransferase 1 associated protein 1; DNMT3A, DNA methyltransferase 3 alpha; DNMT3B, DNA methyltransferase 3 beta; AC, acute control; AS, acute stress; CC, chronic control; CS, chronic stress.

In chicken anterior pituitary glands, changes in expression of DNMTs and DMAP1 genes were measured and compared to those of their controls. DNMT1 and DNMT3B expression were significantly increased by AS (38, and 36%, respectively, *P* < 0.05), but decreased by CS (35, and 41%, respectively, *P* < 0.01) (**Figure 2**). AS and CS did not significantly change DNMT3A expression (*P* = 0.09, *P* = 0.192, respectively). Although the expression level of the DMAP1 was slightly increased (10%) by AS (*P* < 0.05), a significant decrease of 28% occurred following CS (*P* < 0.005), indicating the downregulation of three DNA methylation regulators (DNMT1, DMAP1, and DNMT3A) by CS.

### Effects of Early-Life Nutritional Stress on the Expression of DNA Methylation Regulators in the Anterior Pituitary

**FIGURE 3:**
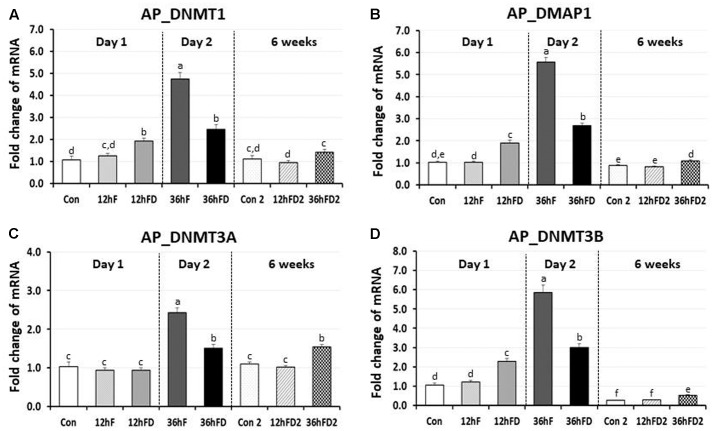
Differential changes in expression of DNA methyltransferases (DNMT1, DNMT3A, and DNMT3B) and DMAP1 in the anterior pituitary by 12 h or 36 h FD (FD; nutritional stress) during the neonatal period. Total RNA from the anterior pituitary was extracted from birds after treatments of 12 h or 36 h FD after post-hatching (Days 1 and 2). Five groups include 0 h controls (Con), 12 h fed controls (12hF), 12 h FD (12hFD), 36 h fed controls (36hFD), and 36 h FD birds (*n* = 14birds/group). At 6 weeks of age, anterior pituitary was sampled from birds which were treated by 12hFD or 36hFD following post-hatching (Days 1 and 2). Birds given food and water *ad libitum* from day 1 through week 6 served as controls (Con2). Relative mRNA levels were quantified and data were set as fold changes of relative expression levels using the ΔΔ*C*t method with GAPDH and β-actin serving as internal controls. Data (mean ± SEM) were expressed from a value set for 1.0 for the 0 h controls (Con) for each gene. Different lower-case letters above the bars denote significant differences (*P* < 0.05) among groups, where a > b > c > d > e > f. c,d is not different from c or d and d,e is not different from d or e. DNMT1, DNA methyltransferase 1; DMAP1, DNA methyltransferase 1 associated protein 1; DNMT3A, DNA methyltransferase 3 alpha; DNMT3B, DNA methyltransferase 3 beta.

In the anterior pituitary gland of FD chicks, DNMT1, DMAP1 and DNMT3B expression were increased 53, 83, and 83% by 12hFD compared to their 12hF controls, respectively (*p* < 0.05) (**Figures 3A,B,D**). In contrast, DNMT1, DMAP1 and DNMT3B decreased 48, 52, and 49% following 36hFD compared to their fed controls 36hF, respectively (*P* < 0.05) (**Figures 3A,B,D**).

DNMT3A showed no significant change in the 12hFD group compared to 12hF control groups (**Figure 3C**). However, similar to the previous three genes, DNMT3A showed a significant decline in gene expression at 36hFD compared to its control group 36hF (**Figure 3C**).

At 6 weeks of age, 12hFD2 groups for all four genes showed no significant changes from their respective control groups (Con2). However, the 36FD2 groups for the DMAP1, DNMT3A and DNMT3B showed consistent and significant increased gene expression compared to Con2 groups (**Figures 3B–D**).

### Effects of Early-Life Nutritional Stress on the Expression of DNA Methylation Regulators and the Glucocorticoid Receptor in Liver

**FIGURE 4:**
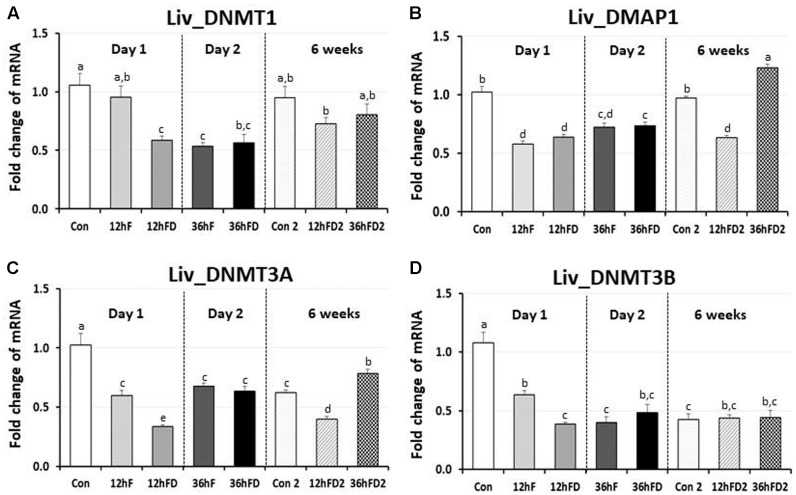
Differential changes in expression of DNA methyltransferases (DNMT1, DNMT3A, and DNMT3B) and DMAP1 in the liver (Liv) following 12 h or 36 h FD (nutritional stress) during the neonatal period. Total RNA from the Liv was extracted from birds after treatments of 12 h or 36 h FD (Days 1 and 2). Five treatment groups included 0 h controls (Con), 12 h fed controls (12hF), 12 h FD (12hFD), 36 h fed controls (36hFD), and 36 h FD birds (*n* = 14 birds/group). At 6 weeks of age, Liv was sampled from birds which were treated by 12hFD or 36hFD following post-hatching (Days 1 and 2). Birds given food and water *ad libitum* from day 1 through week 6 served as controls (Con2). Relative mRNA levels were quantified and data were set as fold changes of relative expression levels using the ΔΔ*C*t method with GAPDH and β-actin serving as internal controls. Data (mean ± SEM) were expressed from a value set for 1.0 for the 0 h controls (Con) for each gene. Different lower-case letters above the bars denote significant differences (*P* < 0.05) among groups, where a > b > c > d > e. a,b is not different from a or b, and b,c is not different from b or c, and c,d is not different from c or d. DNMT1, DNA methyltransferase 1; DMAP1- DNA methyltransferase 1 associated protein 1; DNMT3A, DNA methyltransferase 3 alpha; DNMT3B, DNA methyltransferase 3 beta.

**FIGURE 5:**
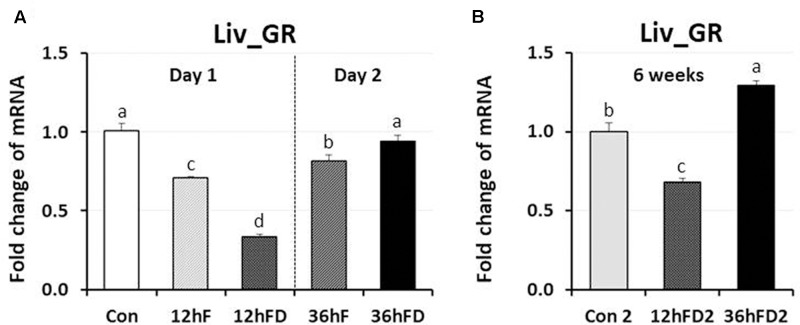
Differential changes in expression of glucocorticoid receptor in the liver (Liv) by 12 h or 36 h FD (nutritional stress) during the neonatal period. **(A)** Total RNA from the Liv was extracted from birds after treatments of 12 h or 36 h FD following post-hatching (Days 1 and 2). Five groups include 0 h controls (Con), 12 h fed controls (12hF), 12 h FD (12hFD), 36 h fed controls (36hFD), and 36 h FD birds (*n* = 14birds/group). **(B)** At 6 weeks of age, Liv was sampled from birds which were treated by 12hFD and 36hFD following post-hatching (Days 1 and 2). Birds given food and water *ad libitum* from day 1 through week 6 served as controls (Con2). Relative mRNA levels were quantified and data were set as fold changes of relative expression levels using the ΔΔ*C*t method with GAPDH and β-actin serving as internal controls. Data (mean ± SEM) were expressed from a value set for 1.0 for the 0 h controls (Con) for each gene. Different lower-case letters above the bars denote significant differences (*P* < 0.05) among groups, where a > b > c > d. DNMT1, DNA methyltransferase 1; DMAP1, DNA methyltransferase 1 associated protein 1; DNMT3A, DNA methyltransferase 3 alpha; DNMT3B, DNA methyltransferase 3 beta.

In the liver of FD stressed birds, DNMT1, DNMT3A, and DNMT3B expression were decreased 39, 44, and 40% by 12hFD compared to their 12hF controls, respectively (*p* < 0.05) (**Figures 4A,C,D**). However, there were no significant changes in DMAP1 expression when compare to their controls (**Figure 4B**). All DNMTs and DMAP1 showed no significant changes in the 36hFD groups compared to 36hF control groups (**Figure 4**). At 6 weeks of age, 12hFD2 groups for DMAP1 and DNMT3A showed significant decreases from their respective control groups (Con2). However, the 36FD2 groups for the DMAP1 and DNMT3A showed significant increased gene expression compared to 12hFD2 groups (**Figures 4B,C**). DNMT1 and DNMT3B showed no significant changes at 6 weeks of age in 12hFD and 36hFD groups compared to their respective controls (Con2). Hepatic glucocorticoid receptor (GR) expression was decreased following 12hFD compared to 12hF controls (**Figure 5A**). In contrast, GR expression was increased by 36hFD compared to their controls (36hF) (*p* < 0.05). At the 6 weeks of age, GR expression patterns persisted compared to their controls (Con2) (**Figure 5**).

## Discussion

### Effects of Immobilization or Nutritional Stress on Plasma CORT

Chronic psychological stress by 1 h immobilization stress for 10 consecutive days induced the down-regulation of CORT levels compared to AS (Kang and Kuenzel, 2014; Nagarajan et al., 2014). In avian species, baseline CORT concentrations are thought to mediate the metabolic needs of daily life and may be increased during periods of elevated metabolic activity (Apfelbeck et al., 2017). In contrast, high CORT concentration occurs when animals are under stress, and is required under certain circumstances to promote life-saving activities for survival (Dallman, 1993; Wingfield et al., 1998). Results in this study indicate that baseline CORT levels are age-dependent and the down-regulation of CORT in chronically stressed birds occurs to reduce responsiveness of the HPA axis for life-saving (**Figure 1**, Krause et al., 2016).

Plasma CORT levels in the 12 h or 36 h FD treated birds were significantly higher compared to their controls. We suggest that the 12hFD group is comparable to the AS immobilization birds, since the latter showed a 3.4-fold increase while the former showed a 2.1-fold increase in CORT. In contrast, the 36hFD group appears comparable to the CS immobilization birds, since the former showed a 1.4-fold increase while latter showed a 1.2-fold increase in CORT. Clearly, even though the 36hFD birds showed the highest CORT levels (**Figure 1B**), the 36hFD birds were stressed three times longer than the 12hFD group, therefore we would expect their CORT levels to be three times higher than the 12hFD group. Their CORT levels, in contrast, were much lower than expected, suggesting an adaptation to their extended period of food restriction, similar to the adaptation of the stress response of birds subjected to CS. However, the CORT results were only suggestive regarding whether the 36 h FD treatment progressed to the CS state or not. Importantly, the significantly reduced expression of DNA methylation regulator genes by both immobilization (AS to CS, **Figure 2**) and FD stress experiments (36hF to 36hFD, **Figure 3**) indicated that 36 h FD is chronic stressor.

### Contrasting Effects of Acute and Chronic Immobilization Stress on the Expression of DNA Methylation Regulators in the Anterior Pituitary

**FIGURE 6:**
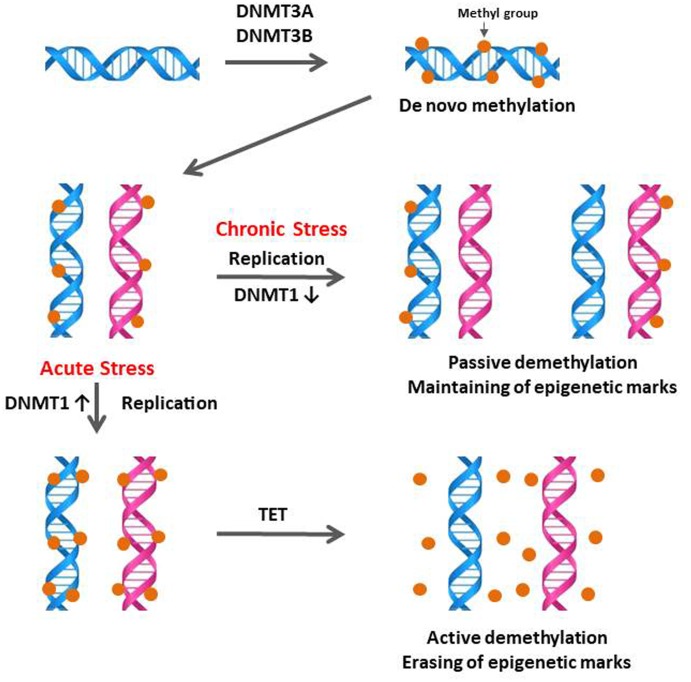
Schematic of the proposed mechanisms of DNA methylation and demethylation involved in acute and chronic stress responses of birds. During initial or acute stress, DNA methylations are caused by the *de novo* DNA methyltransferases DNMT3A and DNMT3B. When DNA replication occurs in the acute stress state, these methyl marks are maintained by the DNMT1, which has preferences for hemi-methylated DNA. If DNMT1 is inhibited by chronic stress, the newly synthesized strand of DNA will not be methylated and results in passive demethylation for maintaining of methylation marks (methyl groups in DNA). In contrast to the passive demethylation, active demethylation in acute stress can erase and remove the DNA methylation marks (methyl groups in DNA) to maintain homeostasis of methylation status. Figure adapted from Wu and Zhang (2010). DNMT1, DNA methyltransferase 1; DMAP1, DNA methyltransferase 1 associated protein 1; DNMT3A, DNA methyltransferase 3 alpha; DNMT3B, DNA methyltransferase 3 beta. TET, Ten–eleven translocation.

DNA methylation is a possible stress-adaptation mechanism involved in the down-regulation of specific genes which are activated by stress (Murgatroyd et al., 2009; Mifsud et al., 2011; Schubeler, 2015) and DNA methylation patterns are not permanent and can be a physiological response to environmental changes (Li and Zhang, 2014). The specific temporal regulation of *de novo* methylation and demethylation is particularly critical for the differentiation and maturation of the central nervous system (CNS) in mammals (Moore et al., 2013). Therefore, the observation of down-regulation of CORT by CS compared to AS (**Figure 1A**) suggested that stress-induced expression of DNA methylation regulators may be increased by acute immobilization stress and decreased by CS in the anterior pituitary. The results of the augmented DNMT1 and DNMT3B expression by acute immobilization stress indicate that, in the chicken anterior pituitary, *de novo* DNA methylation was activated by DNMT3B and it is possible that active demethylation pathway have occurred by activation of DNMT1 resulting in the erasing of epigenetic marks (**Figure 6**), however, these results need to be validated by the actual methylation status study. Importantly these results support past studies showing that reversible DNA methylation by active demethylation is a fundamental epigenetic mechanism and may play a critical role in the regulation of homeostasis for stress (Zannas and West, 2014; Saunderson et al., 2016).

The importance of demethylation has been investigated to a greater extent in recent years as it has become apparent that DNA methylation status can change quite rapidly (Wu and Zhang, 2010; Gong and Zhu, 2011; Mifsud et al., 2011; Cao et al., 2015; Bochtler et al., 2017). The exact biochemical processes involved in DNA demethylation remain unclear but there are a number of mechanisms currently being discussed (Wu and Zhang, 2010; Mifsud et al., 2011). DNA demethylation is characterized as active, passive, or a combination of both. The active demethylation process during reprogramming was reported recently with the finding of Ten–Eleven translocation (TET) enzymes with 5mC oxidase activity and is involved in several physiological responses (Li et al., 2010; Wu and Zhang, 2010; Ge et al., 2014; Guo et al., 2014; Hu et al., 2014). In the current study, down-regulation of DNMT1 expression by CS indicates a possibility of passive demethylation for maintaining epigenetic methylation marks which were established during the AS period (**Figures 2**, **6** in this study; Wu and Zhang, 2010; Saunderson et al., 2016). Passive DNA demethylation is to the elimination of the methyl group from 5mC when DNMT1 activity is decreased or vanished during successional rounds of DNA replication and allows newly incorporated cytosine to remain unmethylated (**Figure 6** in this study; Wu and Zhang, 2010; Sadakierska-Chudy et al., 2015). It’s noteworthy that the passive DNA demethylation mechanism is suggested to be involved in the imperfect maintenance in dividing cells permanently and may cause vulnerability to stress (Zannas and West, 2014; Schubeler, 2015). Therefore, it may be possible that an irreversible phenotypic change of stress-induced genes occurs in the avian anterior pituitary gland via downregulation of DNMT1 induced by CS.

### Tissue Specific Expression of DNA Methylation Regulators Following Early-Life Nutritional Stress and Its Lifelong Effects

In the anterior pituitary, expression patterns of DNA methylation regulators were very similar in the immobilization stressed birds compared to those of the nutritionally stressed birds. Acute immobilization stress and 12 h FD stress stimulated while CS and 36 h FD stress inhibited expressions of DNMT1 and DNMT3B. These results support the previous plasma CORT data, suggesting that the 36 h FD should be regarded as CS. Unique differences in the expression of DNA methylation regulators in the anterior pituitary between immobilization stressed birds and nutritional stressed groups was a significant activation of DMAP1 by 12hFD nutritional stress and a significant inhibition of DNMT3A by the 36hFD stress group compared to controls. Therefore, the results of the current study suggest that the 36hFD treatment should be considered as a chronic nutritional stressor that directly impacted the HPA axis in neonatal chicks.

### A Possible Role of the Hepatic Glucocorticoid Receptor in Long-Term Stress Effects Following Food Deprivation during the Neonatal Period of Birds

Acute stress-induced glucocorticoids (GCs) bind to the GR to form a major complex, the GCs-GR complex. The complex serves as a negative-feedback system to regulate the stress response. It functions by targeting glucocorticoid response elements (GREs) of the stress responsive gene promoters and either directly binds to DNA or tethers onto other DNA-binding transcription factors thereby regulating transcription of its primary target genes (Kuo et al., 2013). Results of the current study showed that acute nutritional stress decreases hepatic GR gene expression, indicating that the suppression of GR expression may be a negative-feedback adaptive mechanism to protect the liver against the potentially damaging effect of nutritional stress. However, GR expression was augmented by 36hFD stress which resulted in higher plasma CORT levels than 12hFD. Hence, a negative feedback system where high CORT levels resulted in downregulation of liver GR in ASed birds (12hFD group) was shown to shift to a positive feedback response between plasma CORT and GR gene expression when food restriction persisted (36hFD group). The shift may suggest a critical change in the animal’s metabolism responsible for adapting to a stressor that has become chronic. One possible mechanism that has been proposed to be critical during neonatal stress adaptation is that AS has been shown to decrease GR gene expression through increased DNA methylation of discrete CpG residues within the promoter of the GR gene, most likely facilitated by DNMT3A (Palma-Gudiel et al., 2015; Mifsud et al., 2017). Of interest, the GR expression patterns persisted when birds were sampled at 6 weeks of age (**Figure 5B**). Other genes that showed persistent, significant increases in gene expression included DNMT3A and DMAP1 in liver and DNMT3A, DNMT3B, DMAP1, and DNMT1 in anterior pituitary. Hence, early-life significant increases in CORT in chicks may result in a potential lifelong, epigenetic effect that could influence the stress response in birds. Limitation of this study is that changes of actual methylation status of the chicken GR promoter were not measured in the response of stress, which requires further study.

## Conclusion

The present study shows that changes in expression of DNA methylation regulators in response to acute and CS are different in the anterior pituitary, suggesting that a passive demethylation mechanism via downregulation of DNMT1 expression may occur following CS in the anterior pituitary of avian species. Results from the early-life nutritional stress experiments demonstrate that expression of DNA methylation regulators respond in a tissue specific manner. Importantly, persistent changes in expression of hepatic GR found at 6 weeks of age suggest that epigenetic changes in hepatic GR expression by early-life nutritional stress may occur by passive DNA demethylation.

## Author Contributions

SK had full access to all the data in the study and take responsibility for the integrity and accuracy of the data. Study concept and design: SK and WK. Acquisition, analysis, or interpretation of data: SK and MM. Drafting and critical revision of the manuscript: SK and WK. Obtained funding: WK, SK, and MM.

## Conflict of Interest Statement

The authors declare that the research was conducted in the absence of any commercial or financial relationships that could be construed as a potential conflict of interest.

## Footnotes

1 http://frodo.wi.mit.edu

## References

[B1] Aman N. A. Nagarajan G. Kang S. W. Hancock M. Kuenzel W. J. (2016). Differential responses of the vasotocin 1a receptor (V1aR) and osmoreceptors to immobilization and osmotic stress in sensory circumventricular organs of the chicken (*Gallus gallus*) brain. *Brain Res.* 1649 67–78. 10.1016/j.brainres.2016.08.028 27559012

[B2] Apfelbeck B. Helm B. Illera J. C. Mortega K. G. Smiddy P. Evans N. P. (2017). Baseline and stress-induced levels of corticosterone in male and female Afrotropical and European temperate stonechats during breeding. *BMC Evol. Biol.* 17:114. 10.1186/s12862-017-0960-9 28532466 PMC5441054

[B3] Armstrong V. L. Rakoczy S. Rojanathammanee L. Brown-Borg H. M. (2014). Expression of DNA methyltransferases is influenced by growth hormone in the long-living Ames dwarf mouse *in vivo* and *in vitro*. *J. Gerontol. A Biol. Sci. Med. Sci.* 69 923–933. 10.1093/gerona/glt133 24201695 PMC4111294

[B4] Baubec T. Colombo D. F. Wirbelauer C. Schmidt J. Burger L. Krebs A. R. (2015). Genomic profiling of DNA methyltransferases reveals a role for DNMT3B in genic methylation. *Nature* 520 243–247. 10.1038/nature14176 25607372

[B5] Bird A. (2002). DNA methylation patterns and epigenetic memory. *Genes. Dev.* 16 6–21. 10.1101/gad.947102 11782440

[B6] Bochtler M. Kolano A. Xu G. L. (2017). DNA demethylation pathways: additional players and regulators. *Bioessays* 39 1–13. 10.1002/bies.201600178 27859411

[B7] Cao H. Wang L. Chen B. Zheng P. He Y. Ding Y. (2015). DNA demethylation upregulated Nrf2 expression in Alzheimer’s disease cellular model. *Front. Aging Neurosci.* 7:244 10.3389/fnagi.2015.00244 PMC4700271 26779013

[B8] Chen Z. X. Riggs A. D. (2011). DNA methylation and demethylation in mammals. *J. Biol. Chem.* 286 18347–18353. 10.1074/jbc.R110.205286 21454628 PMC3099650

[B9] Cheng R. Y. Birely L. A. Lum N. L. Perella C. M. Cherry J. M. Bhat N. K. (2004). Expressions of hepatic genes, especially IGF-binding protein-1 correlating with serum corticosterone in microarray analysis. *J. Mol. Endocrinol.* 32 257–278. 10.1677/jme.0.0320257 14766007

[B10] Choi S. W. Claycombe K. J. Martinez J. A. Friso S. Schalinske K. L. (2013). Nutritional epigenetics: a portal to disease prevention. *Adv. Nutr.* 4 530–532. 10.3945/an.113.004168 24038247 PMC3771139

[B11] Dallman M. F. (1993). Stress update: adaptation of the hypothalamic-pituitary-adrenal axis to chronic stress. *Trends Endocrinol. Metab.* 4 62–69. 10.1016/S1043-2760(05)80017-7 18407136

[B12] Dixon L. M. Sparks N. H. Rutherford K. M. (2016). Early experiences matter: a review of the effects of prenatal environment on offspring characteristics in poultry. *Poult. Sci.* 95 489–499. 10.3382/ps/pev343 26614679 PMC4957487

[B13] Ge L. Zhang R. P. Wan F. Guo D. Y. Wang P. Xiang L. X. (2014). TET2 plays an essential role in erythropoiesis by regulating lineage-specific genes via DNA oxidative demethylation in a zebrafish model. *Mol. Cell. Biol.* 34 989–1002. 10.1128/MCB.01061-13 24396069 PMC3958037

[B14] Ginno P. A. Lott P. L. Christensen H. C. Korf I. Chedin F. (2012). R-loop formation is a distinctive characteristic of unmethylated human CpG island promoters. *Mol. Cell.* 45 814–825. 10.1016/j.molcel.2012.01.017 22387027 PMC3319272

[B15] Gong Z. Zhu J. K. (2011). Active DNA demethylation by oxidation and repair. *Cell Res.* 21 1649–1651. 10.1038/cr.2011.140 21862972 PMC3357985

[B16] Gonzalez-Recio O. Toro M. A. Bach A. (2015). Past, present, and future of epigenetics applied to livestock breeding. *Front. Genet.* 6:305 10.3389/fgene.2015.00305 PMC4585102 26442117

[B17] Guo F. Li X. Liang D. Li T. Zhu P. Guo H. (2014). Active and passive demethylation of male and female pronuclear DNA in the mammalian zygote. *Cell Stem Cell* 15 447–458. 10.1016/j.stem.2014.08.003 25220291

[B18] Head J. A. (2014). Patterns of DNA methylation in animals: an ecotoxicological perspective. *Integr. Comp. Biol.* 54 77–86. 10.1093/icb/icu025 24785828

[B19] Hu X. Zhang L. Mao S. Q. Li Z. Chen J. Zhang R. R. (2014). Tet and TDG mediate DNA demethylation essential for mesenchymal-to-epithelial transition in somatic cell reprogramming. *Cell Stem Cell* 14 512–522. 10.1016/j.stem.2014.01.001 24529596

[B20] Jayanthi S. Kang S. W. Bingham D. Tessaro B. A. Suresh Kumar T. K. Kuenzel W. J. (2014). Identification of antagonists to the vasotocin receptor sub-type 4 (VT4R) involved in stress by molecular modelling and verification using anterior pituitary cells. *J. Biomol. Struct. Dyn.* 32 648–660. 10.1080/07391102.2013.787025 23672311 PMC4035235

[B21] Jeltsch A. Jurkowska R. Z. (2014). New concepts in DNA methylation. *Trends Biochem. Sci.* 39 310–318. 10.1016/j.tibs.2014.05.002 24947342

[B22] Juul-Madsen H. R. Su G. Sorensen P. (2004). Influence of early or late start of first feeding on growth and immune phenotype of broilers. *Br. Poult. Sci.* 45 210–222. 10.1080/00071660410001715812 15222418

[B23] Kang S. W. Kuenzel W. J. (2014). Regulation of gene expression of vasotocin and corticotropin-releasing hormone receptors in the avian anterior pituitary by corticosterone. *Gen. Comp. Endocrinol.* 204 25–32. 10.1016/j.ygcen.2014.04.018 24815884

[B24] Kim M. Costello J. (2017). DNA methylation: an epigenetic mark of cellular memory. *Exp. Mol. Med.* 49:e322. 10.1038/emm.2017.10 28450738 PMC6130213

[B25] Krause J. S. Perez J. H. Chmura H. E. Meddle S. L. Hunt K. E. Gough L. (2016). The stress response is attenuated during inclement weather in parental, but not in pre-parental, Lapland longspurs (*Calcarius lapponicus*) breeding in the Low Artic. *Horm. Behav.* 83 68–74. 10.1016/j.yhbeh.2016.05.018 27215934

[B26] Kuenzel W. J. Kang S. W. Jurkevich A. (2013). Neuroendocrine regulation of stress in birds with an emphasis on vasotocin receptors (VTRs). *Gen. Comp. Endocrinol.* 190 18–23. 10.1016/j.ygcen.2013.02.029 23500673

[B27] Kuo T. Harris C. A. Wang J. C. (2013). Metabolic functions of glucocorticoid receptor in skeletal muscle. *Mol. Cell. Endocrinol.* 380 79–88. 10.1016/j.mce.2013.03.003 23523565 PMC4893778

[B28] Li E. Zhang Y. (2014). DNA methylation in mammals. *Cold Spring Harb. Perspect. Biol.* 6:a019133. 10.1101/cshperspect.a019133 24789823 PMC3996472

[B29] Li Y. Liu Y. Strickland F. M. Richardson B. (2010). Age-dependent decreases in DNA methyltransferase levels and low transmethylation micronutrient levels synergize to promote overexpression of genes implicated in autoimmunity and acute coronary syndromes. *Exp. Gerontol.* 45 312–322. 10.1016/j.exger.2009.12.008 20035856 PMC2838973

[B30] Ludwig A. K. Zhang P. Cardoso M. C. (2016). Modifiers and readers of DNA modifications and their impact on genome structure, expression, and stability in disease. *Front. Genet.* 7:115. 10.3389/fgene.2016.00115 27446199 PMC4914596

[B31] Maniam J. Antoniadis C. Morris M. J. (2014). Early-life stress, HPA axis adaptation, and mechanisms contributing to later health outcomes. *Front. Endocrinol.* 5:73. 10.3389/fendo.2014.00073 24860550 PMC4026717

[B32] Mifsud K. R. Gutierrez-Mecinas M. Trollope A. F. Collins A. Saunderson E. A. Reul J. M. (2011). Epigenetic mechanisms in stress and adaptation. *Brain Behav. Immun.* 25 1305–1315. 10.1016/j.bbi.2011.06.005 21704151

[B33] Mifsud K. R. Saunderson E. A. Spiers H. Carter S. D. Trollope A. F. Mill J. (2017). Rapid down-regulation of glucocorticoid receptor gene expression in the dentate gyrus after acute stress in vivo: role of DNA methylation and microRNA activity. *Neuroendocrinology* 104 157–169. 10.1159/000445875 27054829

[B34] Moore L. D. Le T. Fan G. (2013). DNA methylation and its basic function. *Neuropsychopharmacology* 38 23–38. 10.1038/npp.2012.112 22781841 PMC3521964

[B35] Murdoch B. M. Murdoch G. K. Greenwood S. McKay S. (2016). Nutritional influence on epigenetic marks and effect on livestock production. *Front. Genet.* 7:182. 10.3389/fgene.2016.00182 27822224 PMC5075561

[B36] Murgatroyd C. Patchev A. V. Wu Y. Micale V. Bockmuhl Y. Fischer D. (2009). Dynamic DNA methylation programs persistent adverse effects of early-life stress. *Nat. Neurosci.* 12 1559–1566. 10.1038/nn.2436 19898468

[B37] Muromoto R. Sugiyama K. Takachi A. Imoto S. Noriko S. Yamamoto T. (2004). Physical and functional interactions between Daxx and DNA methyltransferase 1-associated protein, DMAP1. *J. Immunol.* 172 2985–2993. 10.4049/jimmunol.172.5.2985 14978102

[B38] Nagarajan G. Jurkevich A. Kang S. W. Kuenzel W. J. (2017). Anatomical and functional implications of CRH neurons in a septal nucleus of the avian brain: an emphasis on glial-neuronal interaction via V1a receptors *in vitro*. *J. Neuroendocrinol.* 29. 10.1111/jne.12494 28614607

[B39] Nagarajan G. Tessaro B. A. Kang S. W. Kuenzel W. J. (2014). Identification of arginine vasotocin (AVT) neurons activated by acute and chronic restraint stress in the avian septum and anterior diencephalon. *Gen. Comp. Endocrinol.* 202 59–68. 10.1016/j.ygcen.2014.04.012 24780118

[B40] Palma-Gudiel H. Cordova-Palomera A. Leza J. C. Fananas L. (2015). Glucocorticoid receptor gene (NR3C1) methylation processes as mediators of early adversity in stress-related disorders causality: a critical review. *Neurosci. Biobehav. Rev.* 5 520–535. 10.1016/j.neubiorev.2015.05.016 26073068

[B41] Rountree M. R. Bachman K. E. Baylin S. B. (2000). DNMT1 binds HDAC2 and a new co-repressor, DMAP1 to form a complex at replication foci. *Nat. Genet.* 25 269–277. 10.1038/77023 10888872

[B42] Sadakierska-Chudy A. Kostrzewa R. M. Filip M. (2015). A comprehensive view of the epigenetic landscape part I: DNA methylation, passive and active DNA demethylation pathways and histone variants. *Neurotox. Res.* 27 84–97. 10.1007/s12640-014-9497-5 25362550 PMC4286137

[B43] Saunderson E. A. Spiers H. Mifsud K. R. Gutierrez-Mecinas M. Trollope A. F. Shaikh A. (2016). Stress-induced gene expression and behavior are controlled by DNA methylation and methyl donor availability in the dentate gyrus. *Proc. Natl. Acad. Sci. U.S.A.* 113 4830–4835. 10.1073/pnas.1524857113 27078100 PMC4855538

[B44] Schubeler D. (2015). Function and information content of DNA methylation. *Nature* 517 321–326. 10.1038/nature14192 25592537

[B45] Selvam R. Jurkevich A. Kang S. W. Mikhailova M. V. Cornett L. E. Kuenzel W. J. (2013). Distribution of the vasotocin subtype four receptor (VT4R) in the anterior pituitary gland of the chicken, *Gallus gallus*, and its possible role in the avian stress response. *J. Neuroendocrinol.* 25 56–66. 10.1111/j.1365-2826.2012.02370.x 22849330

[B46] Wan J. Oliver V. F. Wang G. Zhu H. Zack D. J. Merbs S. L. (2015). Characterization of tissue-specific differential DNA methylation suggests distinct modes of positive and negative gene expression regulation. *BMC Genomics* 16:49. 10.1186/s12864-015-1271-4 25652663 PMC4331481

[B47] Wingfield J. C. Maney D. L. Breuner C. W. Jacobs D. J. Lynn S. Ramenofsky M. (1998). Ecological bases of hormone-behavior interactions: the “emergency life history stage”. *Am. Zool.* 38 191–206. 10.1006/gcen.1998.7219 10068505

[B48] Wu S. C. Zhang Y. (2010). Active DNA demethylation: many roads lead to Rome. *Nat. Rev. Mol. Cell. Biol.* 11 607–620. 10.1038/nrm2950 20683471 PMC3711520

[B49] Zannas A. S. West A. E. (2014). Epigenetics and the regulation of stress vulnerability and resilience. *Neuroscience* 264 157–170. 10.1016/j.neuroscience.2013.12.003 24333971 PMC3959582

